# Supplementary material for: Prevalence of mortality among mechanically ventilated patients in the intensive care units of Ethiopian hospitals and the associated factors: A systematic review and meta-analysis
Source: PLoS One. 2024 Jul 23;19(7):e0306277. doi: 10.1371/journal.pone.0306277 (PMC11265714; doi:10.1371/journal.pone.0306277)
Supplement: S1 Table — (DOCX) [file pone.0306277.s002.docx]

S1 Table: Logic grid and search strategy for the systematic review of Mortality of Mechanically Ventilated Patients in Intensive Care Units of Ethiopian Hospitals, 2023.

| **Condition** | **Context** | **Population** |
| --- | --- | --- |
| MeSH  Mortality | MeSH  Ethiopia | MeSH  -- |
| Ket terms  Death  Mortality rate  Rate, mortality rates, mortality  Death rate  Rate, death  Rates, death  Associated factors  Predictors  Determinants | Key terms  Intensive Care Units of Ethiopia  Intensive Care Unit of Ethiopia  Unit, Intensive Care of Ethiopia | Free text  Patients who received mechanical ventilation  Patients who received Ventilations, Mechanical  Patients who received Mechanical Ventilations  Patients who received Ventilation, Mechanical |
| **Data Base** | **Searching terms** | **Number of studies** |
| PubMed | (((((((((((mortality) OR (death)) OR (mortality rate)) OR (rate, mortality)) OR (rates, mortality)) OR (death rate)) OR (rate, death)) OR (rates, death)) AND ((((mechanical ventilation) OR (Ventilations, Mechanical)) OR (Mechanical Ventilations)) OR (Ventilation, Mechanical))) AND (((Intensive Care Units) OR (Intensive Care Unit)) OR (Unit, Intensive Care))) AND ((((Patients) OR (Patient)) OR (Clients)) OR (Client))) AND ((Ethiopia) OR (Federal Democratic Republic of Ethiopia)) | 32 |
| Google scholar | (‘mortality’ OR “death’ OR ‘mortality rate’ OR ‘rate, mortality’ OR ‘rates, mortality’ OR ‘death rate’ OR ‘rate, death’ OR ‘rates, death’) AND (‘Associated factors’ OR ‘ Predictors’ OR ‘Determinants’) AND (‘Patients who received mechanical ventilation’ OR ‘Patients who received Ventilations, Mechanical’ OR ‘Patients who received Mechanical Ventilations’ OR ‘Patients who received Ventilation, Mechanical’) AND (‘Intensive Care Units’ OR ‘Intensive Care Unit’ OR ‘Unit, Intensive Care’) AND (‘Ethiopia’ OR ‘Federal Democratic Republic of Ethiopia’) | 1,730 |
| Hinari | ((Mortality) OR (death) OR (death rate) OR (mortality rate)) AND ((associated factors) OR (predictors) OR (determinants)) AND ((mechanically ventilated patients) OR (patients who received mechanical ventilation)) AND ((Intensive Care Units) OR (ICU)) AND (Ethiopia) | 8 |
| Other searches |  | 5 |
| Total retrieved articles |  | 1,775 |
| Number of included studies |  | 22 |
