# Supplementary material for: Prevalence of mortality among mechanically ventilated patients in the intensive care units of Ethiopian hospitals and the associated factors: A systematic review and meta-analysis
Source: PLoS One. 2024 Jul 23;19(7):e0306277. doi: 10.1371/journal.pone.0306277 (PMC11265714; doi:10.1371/journal.pone.0306277)
Supplement: Attachment — Submitted filename: Response to Reviewers.docx [file pone.0306277.s004.docx]

**A point-by-point response to review comments to the author**
Dear Editor and reviewers, we are very grateful for your constructive comments to our submitted manuscript. Your comments are of great value. We have addressed them point by point in the revised manuscript. The revisions are indicated with tracked changes in the revised manuscript. Here below is a table showing point by point responses to your comments.

**Editor’s comments**

Please ensure that your manuscript meets PLOS ONE's style requirements, including those for file naming.

- Thank you for your feedback. It is corrected as recommended.

Please include captions for your Supporting Information files at the end of your manuscript, and update any in-text citations to match accordingly.

- Thank you for your feedback. It is corrected as recommended in the revised version

**Reviewer #1**

I was curious as to why the number of included studies became 22 for the final systematic review and meta-analysis? Based on what did you exclude the last 4 studies from the 26 studies? It would be great if you could clarify that.

- Thank you so much, we have explained it in the revised manuscript on page 6

I would suggest adding legends to the figures

- Thank you, figure legends are incorporated	throughout the whole manuscript

**Reviewer #2**

**Abstract**

Establishing cause-and-effect relationships can be challenging when conducting a systematic review using observational studies. These studies merely observe and record events without manipulating variables. Therefore, it is crucial to explicitly state this limitation in the abstract's conclusion, refraining from making claims that the available evidence cannot substantiate.

- Thank you so much, we really appreciate your comments. It is addressed in the revised manuscript at the conclusion of the abstract section and at the conclusion section of the manuscript on pages	3 & 13

**Methods**

The study's inclusion criteria were carefully designed to be specific and broad enough to cover a wide range of patients. Although the mortality rates between adult and pediatric ICU patients were significantly different, age differences were not considered a distinguishing factor to ensure a comprehensive review that encompasses all relevant studies.

- Thank you in advance, actually we didn’t restrict our inclusion criteria using age category. So, we have revised the inclusion criteria to include a statement “both pediatric and adult ICU-admitted patients” to make it clearer for readers that we had considered age categories on page 5

The study's secondary outcome was not specified or mentioned in the methods sections, making it unclear which factors the systematic review will focus on.

- Dear reviewer, thank you so much. We have addressed it in the revised manuscript on page 5.

**Results**

For subgroup analysis, it may be helpful to consider the study type (prospective cohort, retrospective cohort and cross-sectional) and age differences (adults and pediatrics).

- Thank you so much, sub-group analysis was done using these variables in the revised manuscript on page 8

Why did they choose this publication and sample size to check the cause of heterogeneity of the article?

- Dear reviewer, thank you so much. we use sample size, JBI quality and publication year to run meta-regression analysis. Meta-regression analysis command uses only integer variables and do not allow string variables like region and study design on pages	9-10

The weight of each study was around 4. As I am not a statistician, I wonder how they get similar weights for different studies with varying populations and sample sizes.

- Dear reviewer, thank you for your concerns. Yes, of course, the weight of each study is almost close to 4. This might be because we used a random effects model. Under the random-effects model the goal is not to estimate one true effect, but to estimate the mean of a distribution of effects. Since each study provides information about a different effect size, we want to be sure that all these effect sizes are represented in the summary estimate. This means that we cannot discount a small study by giving it a very small weight. The estimate provided by that study may be imprecise, but it is information about an effect that no other study has estimated. By the same logic we cannot give too much weight to a very large study. Our goal is to estimate the mean effect in a range of studies and we do not want that overall estimate to be overly influenced by any one of them.

The secondary outcome in the Results section should be rewritten. I’m not sure if the OR is adjusted or the raw OR.

- Dear reviewer, thank you for your suggestion. The odds ratio is the pooled odds ratio (adjusted) after meta-analysis of significant variables from individual studies.

**Discussion**

Most of the area is covered. However, the final conclusion needs to be rewritten. The first part concerning prevalence is acceptable, but the second part needs to be reworded.

- Dear reviewer, thank you for your suggestion, we have made revisions in the conclusion on page 13

Finally, thank you so much again!
