# Supplementary material for: Prevalence of mortality among mechanically ventilated patients in the intensive care units of Ethiopian hospitals and the associated factors: A systematic review and meta-analysis
Source: PLoS One. 2024 Jul 23;19(7):e0306277. doi: 10.1371/journal.pone.0306277 (PMC11265714; doi:10.1371/journal.pone.0306277)
Supplement: Attachment — Submitted filename: Response to Reviewers.docx [file pone.0306277.s005.docx]

**Response to Reviewers**

Dear editor and reviewers, thank you for your constructive comments for the second time. We value your comments and we have addressed them point by point in the revised manuscript. The revisions are indicated with tracked changes in the revised manuscript. Here below is a point-by-point response to the reviewers and the editor.

**Journal Requirements**

Please review your reference list to ensure that it is complete and correct. If you have cited papers that have been retracted, please include the rationale for doing so in the manuscript text, or remove these references and replace them with relevant current references. Any changes to the reference list should be mentioned in the rebuttal letter that accompanies your revised manuscript. If you need to cite a retracted article, indicate the article’s retracted status in the References list and also include a citation and full reference for the retraction notice.

- Dear editor, thank you for your comments regarding the referencing style. We have made revisions. All of the references are changed to Vancouver style after seeing the PLOS Submission Guidelines references section on pages 14 – 18. No retracted articles were cited.

**Review Comments to the Author**

**Reviewer #2:** (No Response)

**Reviewer #3**: Suggest to rephrase the title to "Prevalence of mortality among mechanically ventilated patients in the intensive care units of Ethiopian hospitals and the associated factors: A systematic review and meta-analysis"

- Dear reviewer, thank you for your suggestion. The title is rephrased accordingly on pages 1 & 2

Line 85&86: "These include the presence of comorbidities, length of stay on MV, day
and time of ICU admission, Glasgow coma scale (GCS) during admission" please specify the length of stay e.g more than seven days, and the GCS score of 11 and above (for example).

- Dear reviewer we value your comments, revisions are made to make these specific on page 4

line 185: please correct the spelling of heterogeneity

- Dear reviewer, thank you for your comments, the spelling is corrected on page 8

Please check the journal format for abbreviating the word Figure, is it "Fig 2" or "Fig. 2"

- Dear reviewer, thank you for your comments, but according to manuscript body formatting guidelines of the journal figures are labeled as “Fig” not “Fig.” (<https://journals.plos.org/plosone/s/file?id=wjVg/PLOSOne_formatting_sample_main_body.pdf>)

Line 247: please write in full " is not"

- Dear reviewer, thank you for your comments, the word is written in its full form as “is not” on page 11

Finally, thank you in advance for your prompt and constructive feedbacks.

Sincerely,

Temesgen Ayenew (corresponding author)
