# Supplementary material for: Prevalence of mortality among mechanically ventilated patients in the intensive care units of Ethiopian hospitals and the associated factors: A systematic review and meta-analysis
Source: PLoS One. 2024 Jul 23;19(7):e0306277. doi: 10.1371/journal.pone.0306277 (PMC11265714; doi:10.1371/journal.pone.0306277)
Supplement: Attachment — Submitted filename: Author Response to Reviews.docx [file pone.0306277.s006.docx]

**Author’s Response to Reviews**

Dear Reviewer, many thanks to your constructive feedbacks. We have extensively seen the whole manuscript for spelling and grammar errors. Here below is a point-by-point response to the reviewer’s comments.

**Reviewer #3**

1. Please check spelling error throughout the article. I found a few, with in (should be within), and line 186 (spelling for heterogeneity), line 194 (spelling for yields).

Dear reviewer, thank you for your comments, we have made spelling corrections to these words as well as other spelling errors throughout the manuscript. The corrections are indicated with tracked changes.

2. Please check the grammar: I found error in line 272 and 273. based on the references (47& 48), there were two separate studies, therefore should use support (without s).

Dear reviewer, thank you for your comments, we have made grammar checks and made corrections to these sentence and others throughout the article. The corrections are indicated with tracked changes

We really appreciate your feedback

Temesgen Ayenew,

corresponding author
